# Blended Learning on Blood Pressure Measurement: Investigating Two In-Class Strategies in a Flipped Classroom-Like Setting to Teach Pharmacy Students Blood Pressure Measurement Skills

**DOI:** 10.3390/healthcare9070822

**Published:** 2021-06-28

**Authors:** Samieh Farahani, Imaneh Farahani, Maira Anna Deters, Holger Schwender, Bjoern Bengt Burckhardt, Stephanie Laeer

**Affiliations:** 1 Institute of Clinical Pharmacy and Pharmacotherapy, Heinrich Heine University Duesseldorf, Universitaetsstrasse 1, 40225 Duesseldorf, Germany; imaneh.farahani@hhu.de (I.F.); maira.deters@hhu.de (M.A.D.); bjoern.burckhardt@hhu.de (B.B.B.); stephanie.laeer@hhu.de (S.L.); 2 Mathematical Institute, Heinrich Heine University Duesseldorf, Universitaetsstrasse 1, 40225 Duesseldorf, Germany; holger.schwender@hhu.de

**Keywords:** blood pressure measurement, blended learning, flipped classroom, instructional video, pharmacy education, pharmacy students

## Abstract

For reliable blood pressure measurement, various potential sources of inaccuracies need to be considered to avoid incorrect decision-making. Pharmacy students should be sensitized and taught the skill accordingly. One strategy to teach students’ blood pressure measurement skills might be through a blended learning approach in a flipped classroom-like setting. With a randomized two-arm study among pharmacy students in their eighth semester, the required extent of in-class session in the scope of a blended learning approach in a flipped classroom-like setting was evaluated. Participants’ self-confidence and self-perceived proficiency were evaluated through a survey, and participants’ blood pressure measurement performance was assessed by objective structured clinical examination (OSCE). Participants’ satisfaction with, and perception of, the flipped classroom were also surveyed. The extended in-class activities did not result in a significantly higher increase of participants’ OSCE score and self-assessment score when compared to the brief in-class session. Both in-class sessions yielded a significant increase in the OSCE scores as well as in the self-assessment scores. Moreover, the teaching approaches were predominantly well-received by the students. The use of both flipped classroom-like approaches improved pharmacy students’ blood pressure measurement performance, though the brief in-class session was sufficient. Students’ self-confidence/self-perceived proficiency in blood pressure measurement skills increased similarly in both settings.

## 1. Introduction

High systolic blood pressure depicted the leading risk factor worldwide accounting for 10.4 million deaths in 2017 [1], making proper detection, treatment, and control of hypertension crucial [2]. Besides describing evidence-based treatment strategies for hypertension, the 2018 European Society of Cardiology (ESC)/European Society of Hypertension (ESH) guidelines on atrial hypertension highlight pharmacists’ role in the long-term management of hypertension [3]. Pharmacists are well-accessible health care professionals [4] and the literature indicates that pharmacist intervention has a positive impact on the management of arterial hypertension [5,6,7]. The guidelines on the pharmacy-based hypertension management model, developed by EuroPharm Forum and the World Health Organization (WHO) Countrywide Integrated Noncommunicable Diseases Intervention (CINDI) Programme, include blood pressure measurement as one part of the pharmacist’s intervention strategy [8]. Although, at first sight, blood pressure measurement appears to be a simple procedure [9], there are various potential sources of inaccuracies [10] and inaccurate blood pressure measurement can lead to “diagnostic errors and incorrect decision-making and risk assessment” [11]. Consequently, staff training and retraining on proper blood pressure measurement, also with automatic devices, are of great importance to assure accurate blood pressure measurement, which has been underlined by the WHO [11]. Recently in 2019, the Lancet Commission on Hypertension Group highlighted the importance of standardized training and performance assessment of blood pressure measurement skills during healthcare professional education. Furthermore, they emphasized the need for research to “identify the best methods of delivering training” [9]. Usually, no previous practical blood pressure measurement training takes place in our university’s curriculum before clinical pharmacy courses. For proper blood pressure measurement, pharmacy students should have knowledge on the aspects regarding proper equipment and environment for the blood pressure measurement, proper patient preparation, proper blood pressure measurement technique, and proper documentation and providing of blood pressure measurement readings to the patient [12]. Due to the importance of accurate blood pressure measurement for the identification and proper management of hypertension [11], pharmacy students should be sensitized and taught the skill appropriately. A modern strategy to teach students’ performance in blood pressure measurement skills might be the use of a blended learning approach.

Blended learning is considered as “the thoughtful integration of classroom face-to-face learning experiences with online learning experiences” [13]. One benefit of the blended learning approach is that the online component in the form of internet information and communication tools can offer flexibility in terms of place and time [13]. With the development of information technologies, blended learning approaches have been increasingly studied in health professions students’ education and indicating a positive impact on students’ knowledge and skills [14,15,16,17,18,19]. A meta-analysis by Li et al. found that compared to traditional teaching, blended learning significantly improved nursing students’ knowledge and satisfaction. Regarding the analysis of skills, there was no significant difference between blended learning and traditional teaching. However, they found high heterogeneity in the included studies [20]. Regarding students’ confidence in their ability in the respective studied clinical skill (e.g., health assessment skill, counseling skills), studies show that blended learning results in increased confidence [19,21]. However, in a controlled study by Berga et al., the effect on confidence was not significantly different from traditional teaching [21].

A blending learning approach can be implemented in various formats [22,23,24]. One format to realize blended learning is the flipped classroom [23,25]. The framework of the flipped classroom approach was already introduced in 2000 [26,27] and further developed by Bergmann and Sams [25,28]. Definitions regarding flipped classroom vary [29,30,31,32,33,34]. Jensen et al. describe that in a flipped classroom the students are responsible for content attainment before the class and in-class the instructor guides and facilitates concept application. [33]. Flipped classroom approaches can increase instructor–student interaction [28,35] and they can lead to an increased class time efficiency, as the in-class time is used for more student-centered learning activities [36]. The instructor can spend in-class time to guide students in deeper learning processes [37]. The time efforts for developing such an approach amortize over time because the repertoire of materials may only need to be updated [38,39]. From the students’ perspective, students might consider the assigned preparing material for the out-of-class portion as an extra burden [36,40]. The literature indicates the beneficial effect of flipped classroom approaches on blood pressure measurement skills [41,42]. Moreover, flipped classroom approaches have been found to improve outcomes in examination scores and course grades in various other disciplines and skills [35,43,44,45]. However, there is no universal flipped classroom approach [46]. Furthermore, it appears that the design of the flipped classroom influences the differences in the effect of the flipped classroom dependent on the learning domain and learning objective [47,48,49]. Therefore, research evaluating which elements contribute to the efficacy of a flipped classroom approach is needed. [49] However, there is a shortage of comparative studies on the adequate extent or elements of in-class activities of a blended learning approach in a flipped classroom setting for practical clinical skills.

Given the importance of accurate blood pressure measurement [9,11], the purpose of this study was to compare a brief in-class session against an extended in-class session in order to develop an effective blended learning approach in a flipped classroom-like setting for conveying blood pressure measurement skills. It was hypothesized that the extended in-class session combined with a self-instruction video would lead to better blood pressure measurement performance as well as to greater self-confidence/self-perceived proficiency compared to a brief in-class session combined with a self-instruction video.

## 2. Materials and Methods

### 2.1. Objectives

The primary objective was to evaluate whether a self-instruction video (SIV) in combination with an in-class session comprising an objective structured clinical examination (OSCE) with feedback plus additional in-class activities (group B) would lead to better blood pressure measurement performance as compared to SIV in combination with an in-class session comprising an OSCE with feedback only (group A). The blood pressure measurement skills were quantified with an OSCE.

The secondary objectives were to analyze whether an SIV in combination with an in-class session comprising OSCE with feedback plus additional in-class activities (group B) would lead to greater student self-confidence and self-perceived proficiency in blood pressure measurement skills as compared to SIV in combination with an in-class session comprising an OSCE with feedback only (group A). This was measured by a self-assessment survey. Moreover, the satisfaction and perception of the two different groups were assessed using a perception and satisfaction survey.

### 2.2. Study Design and Procedure

This study was approved by the responsible ethics committee (Study Number 2019-729-andere Forschung erstvotierend) and was conducted in the scope of the clinical pharmacy course at the Heinrich Heine University Duesseldorf, Germany. Pharmacy students in the final (eighth) semester of their pharmacy university studies were invited to participate in the study. The students were informed that neither participation nor non-participation in the study would influence students’ passing of this course. The inclusion criterion was the students’ voluntary, written informed consent. Students who had participated in an elective scientific course the semester before, in which the contents and results of a previous study on blood pressure measurement and blood pressure measurement educational approaches were presented and discussed, were excluded to reduce potential bias. The impact of two different flipped classroom-like approaches with different extents of in-class session in the scope of blended learning was investigated in a randomized controlled manner. The control group (group A) participated in an in-class session consisting of an OSCE and feedback (“brief” in-class session), whereas the intervention group’s (group B) in-class session included OSCE and feedback plus additional in-class activities (“extended” in-class session).

**Figure 1:**
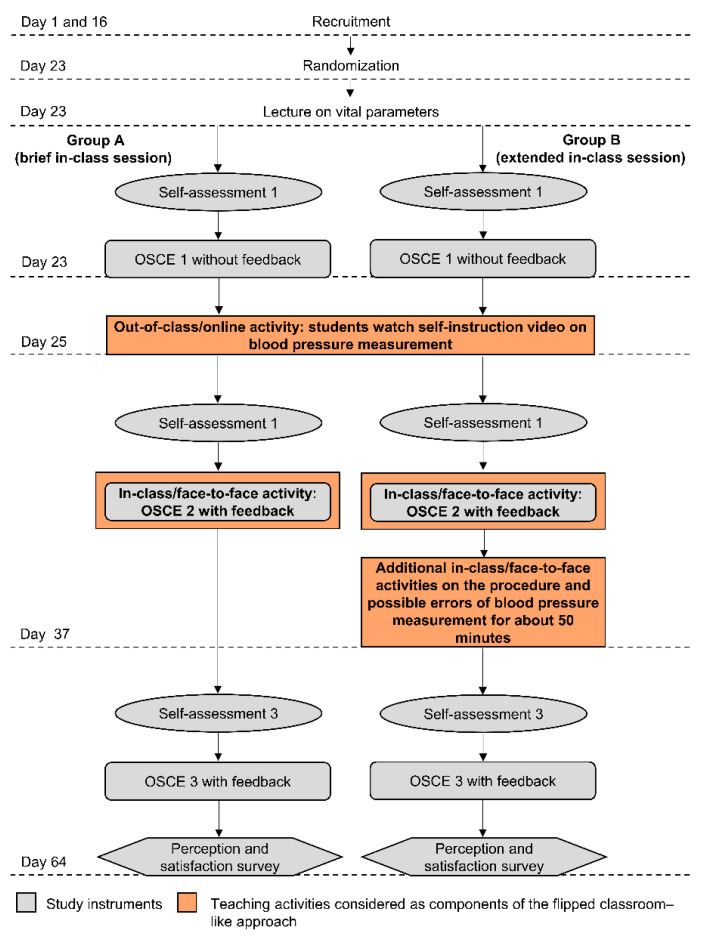
Overview of study design. OSCE = objective structured clinical examination.

The study design and procedure are illustrated in Figure 1. After recruitment, the participants were randomized into two groups—A or B—differing in the extent of in-class activities. On day 23, both groups attended a 60-min lecture on the basic knowledge of hypertension and vital parameters, without presenting the procedure of the oscillometric blood pressure measurement. The lecture is routinely presented every semester during the clinical pharmacy course and was not considered as an actual part of the flipped classroom-like approach as it did not address content regarding the procedure of the oscillometric blood pressure measurement. On the same day, the participants of both groups completed a baseline assessment comprising a self-assessment survey and a subsequent OSCE on blood pressure measurement (assessment 1). Subsequently on day 25, unlimited access to an SIV as out-of-class material was activated for both groups. On day 37, the participants of both groups completed a second self-assessment survey and a subsequent OSCE (assessment 2) to assess the impact of the out-of-class activity (SIV). After completing OSCE 2, each participant of both groups received immediate rater feedback that informed the participants about which steps were missing and/or executed incorrectly using the OSCE checklist (approximately 2 min). The second OSCE and the rater feedback constituted the in-class session of group A. In contrast, group B received additional in-class activities on sources of potential inaccuracies in blood pressure measurement immediately after the second OSCE-session (including feedback) on the same day. In this study setting, OSCE 2 served as a study measurement instrument and as a hands-on exercise for training purposes. The in-class session of each group is detailed in Section 2.3.2. On day 64, both groups participated in a third self-assessment survey and a subsequent OSCE with subsequent rater feedback to assess the impact of the respective in-class session (assessment 3). At the end of this seminar, participants were provided a perception and satisfaction evaluation survey.

### 2.3. Flipped Classroom Approach

The blended learning approach in the format of a flipped classroom-like setting was developed and aimed to convey blood pressure measurement skills to pharmacy students. It was composed of an out-of-class activity, in which a self-instruction video was used for both groups, and an in-class session of an extent dependent on the group.

#### 2.3.1. Out-of-Class Activity

In both study groups, a self-made self-instruction video, customized for pharmacy students, with a total length of 11 min 33 s was provided to the participants. It was meant to give pharmacy students a guide on how to measure the patient’s blood pressure accurately with an oscillometric upper arm device in a community pharmacy setting using a role-play format along with descriptive slides as a self-learning tool [50]. The video was provided on the university’s video platform and was accessible on-demand via the students’ identification data. The video access was activated after assessment 1 and was accessible from this time onward during the complete study for both groups. The participants had the possibility to watch the SIV multiple times, at any time, and pause, replay, and rewind the video according to their preferences. Data regarding participants’ video access was recorded by the university’s multimedia center.

#### 2.3.2. In-Class Session

##### Group with Brief In-Class Session (Group A)

The in-class session of group A (control group) consisted of a single hands-on exercise in the form of OSCE 2 (further described in Section 2.4.1) at assessment 2 and immediate rater feedback.

##### Group with Extended In-Class Session (Group B)

The extended in-class session of group B (intervention group) consisted of OSCE 2 with immediate rater feedback, equivalent to that of group A. However, group B received additional instructor-guided in-class activities for approximately 50 min following assessment 2. For that purpose, the instructors and participants of the intervention group met in a lecture hall. The additional in-class activities started with an assignment in the “thinking-pairing-sharing” format followed by a video case. The thinking-pairing-sharing assignment was based on Frank Lyman’s concept and is a cooperative discussion strategy [51]. The topic of this assignment was potential sources of inaccuracies for the oscillometric as well as auscultatory upper arm blood pressure measurement. At first, each participant was supposed to think about the question individually for approximately 10 min. Thereafter, the participants were told to form groups of two and discuss the question with their partner for approximately 5 min. Finally, the participants were required to share their assumptions with the plenum by raising their hands. During the 20-min sharing phase, an instructor corrected and/or discussed the participants’ solutions, if necessary, and recorded them on the chalkboard. As a second assignment, the participants watched a video, made for this study, of blood pressure measurement in a community pharmacy, with intentional mistakes in the procedure. The participants were asked to identify mistakes in the measurement procedure and share them with the plenum. The video case had a duration of approximately 10 min and the subsequent discussion lasted approximately 5 min.

### 2.4. Data Collection

For the two flipped classroom-like approaches, comprehensive evaluation methods to assess the usefulness and perception of the approaches were applied. In addition to an OSCE checklist on the OSCE performance of the participants, a self-assessment survey, as well as a perception and satisfaction survey, were used.

#### 2.4.1. Objective Structured Clinical Examination

Participants’ blood pressure measurement skills were evaluated by OSCEs. In all three assessments (A1 to A3), the OSCE consisted of one station with the same case, in which each participant was required to take over the role of a pharmacist and perform proper blood pressure measurement for an adult (simulated patient) with an oscillometric upper arm device, pretending to be located in a community pharmacy. During the OSCE encounter, one blood pressure monitor (OMRON M5-Professional-HEM-7001-D), one measuring tape, three different cuff sizes, and writing utensils were provided. The OSCE encounter started with a pre-encounter phase, in which the participants had the possibility to read the candidate instruction, describing the task, followed by an encounter phase of a maximum of 12 min in which the participants were required to perform the blood pressure measurement. If the participant recommended a rest period to the patient, this period was simulated due to time restrictions. In each OSCE encounter, one participant, one simulated patient, and one rater attended. The roles of simulated patients were performed voluntarily by a pool of eight students of their final semester of pharmacy studies, who did not participate in the study and five faculty members. The simulated patients were provided with a script to standardize the role of the simulated patient and were briefed on their task. Four other faculty members assumed the role of raters and assessed students’ blood pressure measurement performances by an OSCE checklist.

#### 2.4.2. OSCE Checklist

The participants’ performance of blood pressure measurement was assessed by OSCE 1, OSCE 2, and OSCE 3, with each participant being assessed by a rater filling in a checklist. The OSCE checklist, taken from a previous study [52], was slightly modified and still consisted of 37 items divided into the sections “general preparation of blood pressure measurement,” “rest period,” “steps of blood pressure measurement,” and “documentation.” The checklist items were weighted equally with one point being awarded if the respective item was fulfilled correctly by the participant and zero points if not, thus a maximum OSCE score of 37 points was achievable. The checklist was based on a literature search that included but was not limited to the standard operating procedure of the Federal Union of German Associations of Pharmacists (ABDA) as of 2017 [53], ESC/ESH Guidelines for the management of arterial hypertension [3], and the American College of Cardiology (ACC)/ American Heart Association (AHA) guideline for the prevention, detection, evaluation, and management of high blood pressure in adults [54].

#### 2.4.3. Self-Assessment Survey

The participants’ self-confidence and self-perceived proficiency in their blood pressure measurement skills were assessed by a self-assessment survey. The survey was the same one as used in a former study [52]. The self-assessment survey was filled in by each participant shortly before OSCE 1, OSCE 2, and OSCE 3 and consisted of 5 items using a 6 point Likert scale (0 = strongly disagree, 1 = disagree, 2 = rather disagree, 3 = rather agree, 4 = agree, 5 = strongly agree), with a maximum of 25 points. Along with the self-assessment survey, participants’ preparation for the blood pressure measurement was collected in each assessment (A1 to A3). Additionally, at A1, the self-assessment survey collected information on participants’ demographics including age, gender, additional education as a pharmaceutical technician assistant, and current or former work in a community pharmacy, and collected data on former experience in blood pressure measurement.

#### 2.4.4. Perception and Satisfaction Survey

Participants’ perception of and satisfaction with the seminar were evaluated by a survey in which they were asked to rate 16 items concerning the seminar and flipped classroom approach by a 6 point Likert scale from “strongly disagree” to “strongly agree.” The participants were also asked to rate the seminar series in the flipped classroom format and to rate the self-instruction video based on the German school grading scale (1 = very good to 6 = insufficient). Further, the survey included free-text questions. The comments the students gave on the free-text questions were grouped into categories, for analysis.

### 2.5. Statistical Analysis

Data from the OSCE checklist and self-assessment survey including demographics and preparation were collected in a pseudonymized way and were anonymized after data analysis. The perception and satisfaction survey was anonymous. Data on participants’ video access was collected via students’ user IDs and was anonymized after analysis. Microsoft Excel [55] was used for data entry, and OriginPro [56] and Microsoft Excel [55] were used for analysis. The participants were randomized to group A or group B using R [57]. A two-sided Mann–Whitney test was applied for baseline comparison of the respective scores between the two groups. The between-group comparison for the score change from assessment 1 to assessment 2 was likewise conducted using a two-sided Mann–Whitney test. A one-sided Mann–Whitney test was applied to assess whether the increase in the respective scores from assessment 2 to assessment 3 was significantly higher in group B (group with extended in-class session) than in group A (group with brief in-class session). A one-sided Mann–Whitney test was applied to assess whether the respective score at assessment 3 was significantly higher in group B than in group A.One-sided Wilcoxon signed-rank tests were used for within-group comparisons. In the perception and satisfaction survey, participants’ German School grade ratings were analyzed using a two-sided Mann–Whitney test. A significance level of alpha = 0.05 was used and asymptotic *p*-values were considered in the following. The *p*-values were not adjusted for multiple testing.

## 3. Results

Forty-six pharmacy students signed the informed consent form and participated in the study, 23 of them were randomized into the group with brief in-class session (group A), and 23 into the group with the extended in-class session (group B). Of these 46 participants, two students from group B and five from group A were excluded from analyses due to non-compliance with the predefined standardized setting, incomplete self-assessment survey data, or absence from an assessment. Thus, 21 participants in group B and 18 participants in group A were included in the analysis of all data, except the perception and satisfaction survey, which was not distributed to participants who missed an assessment. Participants who attended all three assessments but were excluded due to reasons other than the omission of an assessment could not be excluded due to the anonymous character of the perception and satisfaction survey. Participants’ demographics and preparation for OSCE 1, 2, and 3 are depicted in Table 1.

### 3.1. Effect of the Two Flipped Classroom-Like Approaches

The participants’ OSCE score improved similarly in both groups, the group with extended in-class session (group B) and brief in-class session (group A). Also, participants’ self-assessment score improved similarly in both groups. Further details regarding the effect of the two approaches are depicted in the following sections.

#### 3.1.1. OSCE Score

**Figure 2:**
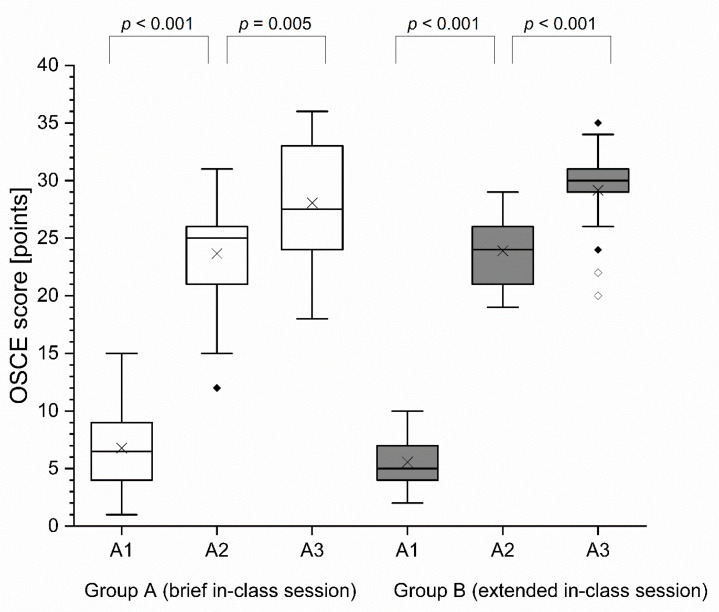
OSCE score per group. Black cross mark (**×**) = mean; horizontal line = median; black diamond (♦) = outlier; white diamond (♢) = extreme value; OSCE = objective structured clinical examination; A1 = assessment 1; A2 = assessment 2; A3 = assessment 3.

At baseline (OSCE 1), the OSCE score did not show a significant difference (*p* = 0.380) between the two groups. From A1 (OSCE 1) to A2 (OSCE 2), the OSCE score increased significantly in both groups (group A: *p* < 0.001; group B: *p* < 0.001). Regarding the increase in OSCE score from A1 to A2, there was no significant difference between the two groups (*p* = 0.213). The OSCE score increased significantly from OSCE 2 (A2) to OSCE 3 (A3) in both groups (group A: *p* = 0.005; group B: *p* < 0.001). Regarding the change in OSCE score from A2 to A3, there was no significantly higher improvement in group B compared to group A (*p* = 0.202). Finally, after the respective teaching approaches, group A achieved at A3 a median OSCE score of 27.5 points (IQR = 9 points) and a mean OSCE-score of 28.06 points (SD = 5.29 points), and group B achieved a median OSCE score of 30 points (IQR = 2 points) and a mean OSCE-score of 29.14 points (SD = 3.65 points) out of a maximum of 37 achievable points of the checklist. At A3 the OSCE score in group B was not significantly higher than the OSCE score in group A (*p* = 0.351). Results of OSCE scores are detailed in Figure 2 and Table 2.

#### 3.1.2. Self-Assessment Score

**Figure 3:**
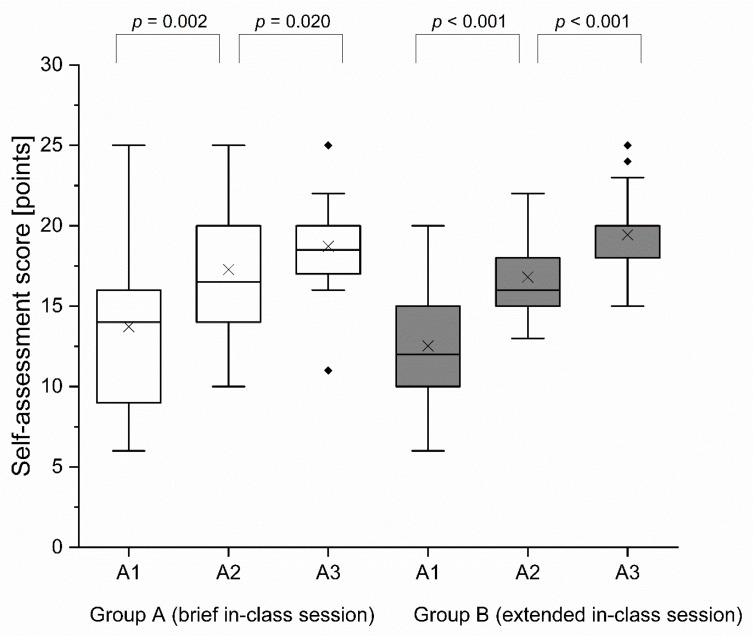
Self-assessment score per group. Black cross mark (×) = mean; horizontal line = median; black diamond (♦) = outlier; A1 = assessment 1; A2 = assessment 2; A3 = assessment 3.

At baseline (self-assessment survey 1), the self-assessment score between the two groups did not show a significant difference (*p* = 0.488). From A1 (self-assessment survey 1) to A2 (self-assessment survey 2), the self-assessment score increased significantly in both groups (group A: *p* = 0.002; group B: *p* < 0.001). Regarding the increase in self-assessment score from A1 to A2, there was no significant difference between the two groups (*p* = 0.178). The self-assessment score increased significantly from A2 to A3 in both groups (group A: *p* = 0.020; group B: *p* < 0.001). Regarding the change in self-assessment score from A2 to A3, there was no significantly higher increase in group B compared to group A (*p* = 0.113). Finally, after the respective teaching approach, group A achieved at A3 a median self-assessment score of 18.5 points (IQR = 3 points) and a mean self-assessment score of 18.72 points (SD = 2.91 points), and group B achieved a median self-assessment score of 20 points (IQR = 2 points) and mean self-assessment score of 19.43 points (SD = 2.77 points) out of a maximum of 25 achievable points of the self-assessment survey. At A3 the self-assessment score in group B was not significantly higher than the self-assessment score in group A (*p* = 0.261). Results of self-assessment scores are detailed in Figure 3 and Table 2.

#### 3.1.3. Perception and Satisfaction Survey

In total, 38 participants returned the perception and satisfaction survey. One survey was excluded because its group allocation was not clearly evident. Thus, 21 surveys from the group with brief in-class session (group A) and 16 from the group with extended in-class session (group B) were included in the analysis. As not all of these participants filled in the survey completely, the total number of responses varied depending on the item. Generally, the majority of both groups indicated an interest in the seminar series on blood pressure measurement (item 1). All participants of both groups agreed (rather agree, agree, or strongly agree) that they felt better prepared for performing correct blood pressure measurement on a real patient in the community pharmacy after the seminar series. The SIV was rated with a mean German school grade of 1.90 (SD = 0.89) and median of 2 (IQR = 1) by group A (*n* = 21) and a mean German school grade of 1.88 (SD = 0.72) and a median of 2 (IQR = 0.5) by group B (*n* = 16) with no significant difference between the two groups (*p* = 1). The seminar series in the form of flipped classroom model was rated with a mean German school grade of 2.3 (SD = 1.22) and median of 2 (IQR = 1.5) by the group with brief in-class session (*n* = 20) and a mean German school grade of 2.44 (SD = 1.03) and a median of 2 (IQR = 1) by the group with extended in-class session (*n* = 16), with no significant difference between the two groups (*p* = 0.519). Further results regarding the perception and satisfaction survey are depicted in Table 3 and Table 4.

#### 3.1.4. Video Access

Data regarding the video access provided by the multimedia center were inconsistent. Nevertheless, for analysis, a total of 103 video hits was used, which was most traceable by additional details provided in the report. Thus, the analysis was carried out based on 103 video hits. Subsequent excluding of the 7 excluded participants acesses and the access by website administrator resulted in 83 video accesses. Before the second OSCE (A2), each participant had accessed the video at least one time (video access mean = 1.69; SD = 0.77) (video access median = 2; IQR = 1). For the third OSCE (A3), the video was not accessed by every participant, leading to a mean video access of 0.44 (SD = 0.60) (median video access = 0; IQR = 1). In particular, for the third OSCE, five students of group A had accessed the video once and one student twice. In group B, eight students had accessed the video once and one student twice. These numbers should be taken with caution as they could include instances of participants only clicking on the video without watching it and/or watching parts of the video multiple times. Furthermore, students might have taken notes on the video and read their notes instead of rewatching the video or several students might have watched the video via one student user ID. These aspects might also be the reason why the counts of video views self-reported by the students (Table 1) do not comply with this video access data.

## 4. Discussion

This study developed an effective teaching approach for conveying blood pressure measurement skills to pharmacy students. This study contributes to the literature by providing data on effective and adequate teaching approaches for blood pressure measurement, as suggested in the literature [9]. A blended learning approach that combines a valuable self-instruction video with a hands-on exercise and subsequent rater feedback in a flipped classroom-like setting allowed students to acquire comprehensive blood pressure measurement skills. However, the hypothesis that we proposed above, was not supported by the present results, as the additional in-class activities did not result in a significantly higher increase in participants’ blood pressure measurement skills or self-confidence/self-perceived proficiency. The flipped classroom approaches were predominantly well accepted in the surveyed students.

In this study, it was found that the blended learning approach consisting of a self-instruction video and hands-on exercise with feedback in a flipped classroom-like setting was optimal to improve pharmacy students’ blood pressure measurement skills. Both the brief and the extended in-class strategy led to a significant increase in blood pressure measurement performance from OSCE 2 to OSCE 3, with no significant higher improvement in the group with the extended in-class session compared to the group with the brief in-class session. Based on these results, we suggest that an in-class session comprising a hands-on exercise in the form of an OSCE with feedback, in combination with a self-instruction video as out-of-class activity, in the scope of a flipped classroom-like approach was sufficient to convey blood pressure measurement skills to pharmacy students. Additional in-class activities on typical errors in blood pressure measurement did not improve their performance further. Moreover, this result underlines the importance and benefit of feedback for students to foster clinical skills as described in the literature [58,59,60,61,62,63]. With the omission of the additional in-class activities, the developed flipped classroom-like approach is efficient, as it leads to saving time and staff while maintaining comparable outcomes.

Our study contributes to the previous literature regarding flipped classroom-like approaches on blood pressure measurement [41,42], as in our design we compared two flipped classroom-like approaches. Prescott et al. compared a traditional approach and a blended-learning model consisting of a flipped classroom format for pharmacy students in a patient assessment course, in which blood pressure measurement was one of the topics. They found that the blended-learning model group performed better in blood pressure measurement than the traditional course group [41]. Bachur et al. found that after undergoing a class with active methodologies as a teaching strategy in the scope of an inverted classroom-like model, the medical and physiology students’ knowledge on and performance in blood pressure measurement improved significantly. In line with our study, the students used an oscillometric blood pressure measurement device [42]. Their focus was on active learning activities in the class rather than the pre-class session. In their study, they applied a single-group design with pre-post-assessment without a control group [42]. In contrast to that, our study aimed to compare two in-class strategies in the scope of a flipped-classroom-like approach in pharmacy students by using two groups and in a pre-post-design. The design of our study complements previous approaches in the literature and contributes to active research in the context of flipped classroom approaches. To the best of our knowledge, there is no study comparing different in-class strategies of a flipped classroom-like approach for teaching blood pressure measurement skills in pharmacy students. Both, Bachur and colleagues and our approaches achieved comparable post-training scores in performance (67.48% Bachur et al. vs 74.32% in group A of the presented study). However, in the study of Bachur et al., blood pressure measurement competence was already theoretically and practically taught in the curriculum before the conduct of their study [42], while our students were taught blood pressure measurement practically in their pharmacy studies for the first time. In our study, the hands-on in-class activity in the form of OSCE 2 was conducted by every student individually, but with instructor–student interaction as the students were provided with feedback by the instructor. Moreover, further interaction was present as the student performed with a simulated patient who was a peer or faculty member. The additional in-class activity in our intervention group comprised instructor-guided activities as well as working in pairs. Consequently, the in-class activities of both groups in our study included active learning and were interactive, which might be a factor contributing to the efficacy of our flipped-classroom approaches, as research indicates that active learning is a pivotal factor for the efficacy of flipped classroom approaches [33].

We assume, in line with other authors, that one important premise for the flipped classroom to be effective is that the students use the provided pre-class activities [64,65,66]. In the literature, the use of graded in-class quizzes or graded homework as incentives for using the pre-class activity is discussed [66,67,68]. Although the second OSCE (as well as the other two OSCEs) in our study had a low-stakes structure with students’ performance having no impact on passing this course, knowing that OSCE 2 would be executed might have incited the participants to engage with the self-instruction video. Finally, it was found that, for OSCE 2, the video was accessed in mean 1.69 times per participant. However, data regarding participants’ video access should be taken with caution, as they could include instances of participants only clicking on the video without watching it and/or watching parts of the video multiple times. Furthermore, students may have taken notes while watching the video the first time and read their notes instead of watching the video again.

Regarding the students’ self-confidence and self-perceived proficiency, the brief in-class activity comprising an OSCE with feedback in combination with a self-instruction video as out-of-class material was found to improve students’ self-confidence significantly. Additional activities on typical errors in blood pressure measurement (extended in-class activity) did not improve students’ self-confidence significantly further. The positive impact of the flipped classroom approach on students’ self-confidence was also reported in a pre-post study by Liu et al. [69]. In line with Liu and colleagues’ findings, in our study participants’ self-confidence and self-perceived proficiency increased significantly. This indicates that our out-of-class activity, as well as the in-class session of both groups, contributed to the promotion of the students’ self-perceived proficiency and confidence. Furthermore, these findings imply that the chosen format of extended in-class session was not superior in terms of participants’ self-confidence. The perception and satisfaction survey revealed that our flipped classroom-like approach was predominantly well received among the students. Both the group with the brief in-class session (71.43%) and the group with the extended in-class session (80%) agreed (rather agree, agree, or strongly agree) that the flipped classroom model should be incorporated in the future pharmacy education. The remaining portion of participants who seemed to show reluctance might have considered the pre-class activities as extra work and increasing their workload, indicated in the literature [39,70,71]. Moreover, students might need time to adjust to the new teaching modality [72].

The approach investigated in this study required the physical attendance of students and instructors simultaneously during the in-class session. Another possible approach might be to set up this seminar completely in a distance education design, which is highly relevant during the COVID-19 pandemic as in-person sessions are limited. For that purpose, given that the students are provided with the equipment, each student could record their execution of the respective skill wherever convenient to them and the instructor could evaluate and provide feedback either synchronously or asynchronously. Moreover, this might be an option to retrain pharmacists in their community pharmacy environment. However, the efficacy of the method and the students’ satisfaction need to be evaluated before implementation.

Our study has some limitations, including intra- and interrater variability, which could potentially have influenced the results bidirectionally. We intended to reduce these variabilities by training the raters beforehand and having each participant being assessed by the same rater for all OSCE assessments. Moreover, for performance evaluation, the raters used a checklist with items described in detail. Due to these controlled procedures, we conjecture that the impact of these limitations on the OSCE performance was low. Moreover, variations in the simulation patients’ acting performance may have occurred. We aimed to standardize the actors’ performance by briefing the simulated actors and providing a script. Therefore, we classify the impact of this potential bias on the OSCE performance as negligible. Although the participants were instructed to not disclose information regarding the OSCEs and activities, it cannot be completely excluded that information might have been exchanged between the study groups.

## 5. Conclusions

In this study, the use of both flipped classroom-like models improved pharmacy students’ blood pressure measurement performance and increased students’ self-perceived proficiency and confidence in blood pressure measurement skills. Furthermore, the findings indicate that in the case of acquiring oscillometric blood pressure measurement skills, a brief in-class session was sufficient. Considering resources and outcomes, in the future, a complete conversion of the training to a fully online approach (i.e., self-instruction video combined with an online OSCE) could be also a promising strategy.

## Figures and Tables

**Table 1:** Demographics of participants.

	Group A (Brief In-Class Session) (*n* = 18)	Group B (Extended In-Class Session) (*n* = 21)
**OSCE 1**		
**1.1 Age in years ** **^a^**		
Median (IQR)	24 (4)	23 (3)
Mean (SD)	25.72 (4.53)	24.4 (3.19)
**1.2. Gender**		
Female, *n* (%)	12 (66.67)	16 (76.19)
Male, *n* (%)	6 (33.33)	5 (23.81)
**1.3. Training as a pharmaceutical technical assistant**		
Yes, *n* (%)	3 (16.67)	2 (9.52)
No, *n* (%)	15 (83.33)	19 (90.48)
**1.4. Currently or formerly worked in a community pharmacy ** **^b^**		
Yes, *n* (%)	8 (44.44)	4 (19.05)
No, *n* (%)	10 (55.56)	17 (80.95)
**1.5. Have measured BP by myself for the first time in my life**		
Yes, *n* (%)	3 (16.67)	6 (28.57)
No, *n* (%)	15 (83.33)	15 (71.43)
**1.6. If answered item 1.5 with no: What kind of blood pressure measurement device/s have you already used to measure the BP? ^c^**		
Upper arm blood pressure monitor	11 (73.33)	13 (92.86)
Wrist blood pressure monitor	10 (66.67)	8 (57.14)
Blood pressure measurement device with stethoscope	5 (33.33)	3 (21.43)
**1.7. Preparation for BP measurement task ** **^d^**		
Yes, *n* (%)	0 (0)	0 (0)
No, *n* (%)	18 (100)	20 (100)
**OSCE 2**		
**2.1. Preparation for BP measurement task**		
Yes, *n* (%)	18 (100)	21 (100)
No, *n* (%)	0 (0)	0 (0)
**2.2. If item 2.1 was answered yes: Did you prepare yourself with the self-instruction video from the university’s video platform?**		
Yes, *n* (%)	18 (100)	21 (100)
No, *n* (%)	0 (0)	0 (0)
**2.3. If item 2.1 was answered yes: Did you use other materials for preparation in addition to the video? ** **^e^**		
Yes, *n* (%)	0 (0)	0 (0)
No, *n* (%)	18 (100)	21 (100)
**OSCE 3**		
**3.1. Preparation for BP measurement task ** **^f^**		
Yes, *n* (%)	15 (83.33)	19 (90.48)
No, *n* (%)	3 (16.67)	2 (9.52)
**3.2. If item 3.1 was answered yes: Did you prepare yourself with the self-instruction video from the university’s video platform?**		
Yes, *n* (%)	15 (100)	18 (94.74)
No, *n* (%)	0 (0)	1 (5.26)
**3.3. If item 3.1 was answered yes: Did you use other materials for preparation in addition to the video? ** **^e,g^**		
Yes, *n* (%)	0 (0)	0 (0)
No, *n* (%)	15 (100)	19 (100)

^a^ Out of n = 20 in group B, as one participant of group B did not fill in the item for age. ^b^ Excluding mandatory internship during the pharmacy studies. ^c^ Although 15 participants of group B answered item 1.5 “Have measured BP by myself for the first time in my life” with “no,” only 14 participants of group B responded to item 1.6. In group A, n = 15 for item 1.6. ^d^ One participant in group B did not respond to item 1.7, resulting in 20 responses in group B for item 1.7. The survey also included item 1.8: “If item 1.7 is answered with yes: How and how long did you prepare for the blood pressure measurement?” As no participant of either group responded “yes,” this item is not listed here. ^e^ The survey at both assessment 2 and assessment 3 also included item 2.4 or 3.4, respectively “If item 2.3/3.3 is answered “yes”: Which additional material did you use?” One participant of group A commented on that item for both assessment 2 and assessment 3, that he/she read his/her notes on the video, while responded to item 2.3 and 3.3 with “no.” ^f^ In group B, one participant responded to item 3.1 with “yes”, while responding to item 3.2 and 3.3 with “no.” ^g^ In group B, the number of participants for item 3.3 was corrected by the participants, who crossed yes and commented that the additional preparation was the extended in-class session. OSCE = objective structured clinical examination; IQR = interquartile range; SD = standard deviation; BP = blood pressure.

**Table 2:** Changes in OSCE score and self-assessment score.

Instrument	Group	Change in Score between A1 and A2 in Points		*p*-Value ^a^	Change in Score between A2 and A3 in Points		*p*-Value ^b^
		Mean (SD)	Median (IQR)		Mean (SD)	Median (IQR)	
OSCE checklist	Group A (*n* = 18)	16.89 (3.88)	16.5 (5)	0.213	4.39 (5.74)	2 (7)	0.202
	Group B (*n* = 21)	18.33 (4.27)	19 (5)		5.24 (4.07)	5 (6)	
Self-assessment survey	Group A (*n* = 18)	3.56 (4.93)	3 (6)	0.178	1.44 (2.64)	1 (4)	0.113
	Group B (*n* = 21)	4.29 (3.26)	5 (3)		2.62 (2.50)	2 (3)	

^a^ A two-sided Mann–Whitney test with alpha = 0.05 was used to assess whether there is a significant difference between groups. ^b^ A one-sided Mann–Whitney test with alpha = 0.05 was used to assess whether the increase in group B (extended in-class session) is significantly higher as compared to group A (brief in-class session). A1 = assessment 1; A2 = assessment 2; A3 = assessment 3; Group A = brief in-class session; Group B = extended in-class session; OSCE = objective structured clinical examination; SD = standard deviation; IQR = interquartile range.

**Table 3:** Results of the perception and satisfaction survey.

	Proportion of Responses, *n* (%)					
	Strongly Disagree	Disagree	Rather Disagree	Rather Agree	Agree	Strongly Agree
I found the seminar series on BP measurement interesting.						
Group A; *n* = 21	0 (0)	1 (4.76)	2 (9.52)	6 (28.57)	7 (33.33)	5 (23.81)
Group B; *n* = 16	0 (0)	0 (0)	2 (12.5)	5 (31.25)	6 (37.5)	3 (18.75)
During the OSCEs/simulations, I was able to determine my strengths and weaknesses in BP measurement.						
Group A; *n* = 21	0 (0)	0 (0)	1 (4.76)	3 (14.29)	6 (28.57)	11 (52.38)
Group B; *n* = 16	0 (0)	0 (0)	0 (0)	0 (0)	9 (56.25)	7 (43.75)
The OSCEs/simulations enabled me to apply the knowledge and skills I gained during the instruction video and the in-class phase.						
Group A; *n* = 21	0 (0)	0 (0)	0 (0)	2 (9.52)	13 (61.90)	6 (28.57)
Group B; *n* = 16	0 (0)	0 (0)	0 (0)	0 (0)	10 (62.5)	6 (37.5)
After this seminar series, I feel better prepared for the correct BP measurement in the community pharmacy on real patients.						
Group A; *n* = 21	0 (0)	0 (0)	0 (0)	0 (0)	11 (52.38)	10 (47.62)
Group B; *n* = 16	0 (0)	0 (0)	0 (0)	3 (18.75)	5 (31.25)	8 (50)
The instruction video was helpful in conveying knowledge about measuring BP.						
Group A; *n* = 21	0 (0)	0 (0)	1 (4.76)	3 (14.29)	8 (38.10)	9 (42.86)
Group B; *n* = 16	0 (0)	0 (0)	0 (0)	2 (12.5)	7 (43.75)	7 (43.75)
The instruction video was helpful in improving my practical BP measurement skills.						
Group A; *n* = 21	0 (0)	0 (0)	0 (0)	6 (28.57)	5 (23.81)	10 (47.62)
Group B; *n* = 16	0 (0)	0 (0)	0 (0)	4 (25)	6 (37.5)	6 (37.5)
I had technical problems accessing or playing the instruction video.						
Group A; *n* = 20	17 (85)	1 (5)	0 (0)	1 (5)	1 (5)	0 (0)
Group B; *n* = 16	14 (87.5)	2 (12.5)	0 (0)	0 (0)	0 (0)	0 (0)
The in-class phase was helpful to improve my understanding of BP measurement.						
Group A; *n* = 20	0 (0)	0 (0)	1 (5)	3 (15)	10 (50)	6 (30)
Group B; *n* = 16	0 (0)	2 (12.5)	1 (6.25)	5 (31.25)	6 (37.5)	2 (12.5)
The in-class phase was helpful in improving my practical BP measurement skills.						
Group A; *n* = 21	0 (0)	0 (0)	1 (4.76)	3 (14.29)	8 (38.10)	9 (42.86)
Group B; *n* = 16	0 (0)	3 (18.75)	1 (6.25)	3 (18.75)	6 (37.5)	3 (18.75)
I prefer the instruction video on its own to train the competence of BP measurement.						
Group A; *n* = 21	3 (14.29)	6 (28.57)	4 (19.05)	4 (19.05)	2 (9.52)	2 (9.52)
Group B; *n* = 16	1 (6.25)	2 (12.5)	6 (37.5)	2 (12.5)	3 (18.75)	2 (12.5)
I prefer the active in-class phase on its own to train the competence of BP measurement.						
Group A; *n* = 21	3 (14.29)	6 (28.57)	4 (19.05)	6 (28.57)	2 (9.52)	0 (0)
Group B; *n* = 16	2 (12.5)	4 (25)	6 (37.5)	3 (18.75)	1 (6.25)	0 (0)
I prefer the combination of instruction video with the active in-class phase undertaken in this study to train the competence of measuring BP.						
Group A; *n* = 21	0 (0)	1 (4.76)	2 (9.52)	3 (14.29)	10 (47.62)	5 (23.81)
Group B; *n* = 16	0 (0)	1 (6.25)	1 (6.25)	5 (31.25)	4 (25)	5 (31.25)
In the future, instruction videos should be included in pharmacy teaching.						
Group A; *n* = 21	0 (0)	1 (4.76)	1 (4.76)	2 (9.52)	9 (42.86)	8 (38.10)
Group B; *n* = 16	0 (0)	0 (0)	0 (0)	2 (12.5)	9 (56.25)	5 (31.25)
OSCEs/simulations about BP measurement are superfluous because one can do nothing wrong with the BP measurement.						
Group A; *n* = 21	13 (61.90)	5 (23.81)	3 (14.29)	0 (0)	0 (0)	0 (0)
Group B; *n* = 16	13 (81.25)	3 (18.75)	0 (0)	0 (0)	0 (0)	0 (0)
In the future, OSCEs/simulations should be included as a regular part of the clinical pharmacy course to train practical skills (such as BP measurement).						
Group A; *n* = 21	0 (0)	1 (4.76)	1 (4.76)	4 (19.05)	11 (52.38)	4 (19.05)
Group B; *n* = 16	0 (0)	0 (0)	0 (0)	5 (31.25)	4 (25)	7 (43.75)
In the future, the “flipped classroom model” should be included in pharmacy teaching.						
Group A; *n* = 21	1 (4.76)	2 (9.52)	3 (14.29)	9 (42.86)	5 (23.81)	1 (4.76)
Group B; *n* = 15	0 (0)	0 (0)	3 (20)	5 (33.33)	4 (26.67)	3 (20)

BP = blood pressure, OSCE = objective structured clinical examination; Group A = brief in-class session; Group B = extended in-class session.

**Table 4:** Exemplary topics of comments on free-text questions/items of the perception and satisfaction survey.

Free-Text Item	Group	Topics
What did you particularly like about the series of seminars in the “flipped classroom model”?	Group A (brief in-class session)	practical application
		repeating several times to consolidate knowledge
		possibility to watch video in your own pace
	Group B (extended in-class session)	practical application
		linking of theory and practice
		no required attendance during self-learning phase (out of class activities)
I would change the following on the on the seminar series in the format of “flipped classroom model”	Group A (brief in-class session	better time management for in-class activities
		request for more practical OSCEs for training purposes like that in the pharmacy studies
		too fast pace
	Group B (extended in-class session)	better time management
		smaller group size and room size during the additional in-class activities regarding the potential mistakes
		seminar regarding the potential mistakes was not particularly helpful

The three most frequent topics of the comments are shown for each item per group. If topics appeared with equal frequency, one topic was chosen. OSCE = objective structured clinical examination.

## Data Availability

The dataset presented in this study is available from the corresponding author on reasonable request.
